# Morphological analysis of coronoid process shape variations in adult human mandibles

**DOI:** 10.6026/973206300212031

**Published:** 2025-07-31

**Authors:** Urmila Sinha, Ajay Kumar Patel, Ruchi Ratnesh, Vishakha Sontakke, Deepa Devadas, Sanjay Kumar

**Affiliations:** 1 Department of Anatomy, All India Institute of Medical Sciences, Deoghar, Jharkhand, India; 2 Department of Anatomy, Banaras Hindu University, Varanasi, Uttar Pradesh, India; 3 Department of Anaesthesiology, All India Institute of Medical Sciences, Deoghar, Jharkhand, India

**Keywords:** Coronoid process, mandible, morphological variation, triangular shape, anatomical study, forensic anthropology

## Abstract

Change in the shape of the coronoid process on 80 dried adult human mandibles is of interest. Data shows that 47.5% of the cases involved triangular coronoid processes, 35% had rounded shapes and 17.5% had hook-shaped coronoid processes. Eight out of ten cases showed bilateral symmetry. Most of the furnishings took a triangular shape. These results are important for identifying people after an accident.

## Background:

As the largest and toughest facial bone, the human mandible supports eating, speaking and protecting the face's appearance. The mandible has a flat body, with two vertical rami on either side, each having the condylar and coronoid processes [1]. Among them, the coronoid process is most important, since it attaches the temporalis muscle, a major muscle used for chewing. The attachment helps the mandible go up and down, so the important coronoid process is involved in chewing, biting and similar activities [2]. Found on the front of the mandibular ramus, the coronoid process juts upwards with some degree of variation. There are clear differences in the body's structure which have drawn interest from clinical anatomy, forensic anthropology and evolutionary biology [3, 4]. A mix of genes, hard chewing and the body's responses to food and chewing are considered to cause these variations [5]. Because eating fibrous foods means chewing vigorously, those foods may strengthen the jaw muscles and increase the size of the prominent coronoid processes [6]. Many variations in how the coronoid process is classified morphologically have been suggested. Triangular types are described as poking, rounded types as softly curved and the hook-shaped type is bent backward, much like a hook [7]. Triangular forms are the most commonly reported, with high numbers coming from various populations [8, 9]. Although these anatomical variations are seemingly small, they are important during various operations, mainly those involving the temporomandibular joint (TMJ), surgeries to straighten teeth and reconstructions or donor grafts [10, 11]. The radiance process on the mandible, along with its general structure, is highly useful in helping the forensic identification of human remains. Due to the robustness of mandibular features, anthropologists regularly use them for judging a person's age, gender and ethnicity when other bones in the skeleton are missing or broken [12, 13]. Understanding how mandibular forms have shifted has helped explain evolution and allowed researchers to differentiate groups, since what people ate and the activities required for chewing have evolved over time [14, 15]. Very few studies have been done on the coronoid process in different regions of the Indian population, which is diverse in genetics, diet and environment. Therefore, it is of interest to investigate the variation in the form of the coronoid process in adult human mandibles. Sorting and measuring the different skull types observed in this study is meant to support anatomists, maxillofacial surgeons, anthropologists and forensic experts, while also having applications in research and medical practice.

## Materials and Methods:

Everything in this analysis was authorized by the appropriate authority before the research was conducted to guarantee it followed approved procedures. Following this, the study was conducted in the Department of Anatomy at All India Institute of Medical Sciences, Deoghar, Jharkhand, India, and Banaras Hindu University, Varanasi over one year as an observational analysis. Eighty adult dry human mandibles were selected from the department's osteology collection. We chose these specimens because their age and sex were uncertain, and we wanted those that were anatomically complete and well-preserved. Any mandibles with visible disorders, changed condition or accidental damage after death were eliminated to avoid bias in analysis. A detailed examination of the macroscopic appearance of the right and left coronoid processes was performed on all mandibles. Because of specific research, these bony projections, found on the front of the mandibular ramus, were grouped as triangular, rounded or hook-shaped in shape. Classification was determined solely by visual examination of the neck, without the need for any radiological tools, to maintain an old-style approach. To prevent bias in recording and to increase the classifier's reliability, two different evaluators reviewed the shape of each coronoid process. If their opinions differed, a review and discussion helped to settle the disagreement. The researchers recorded whether the coronoid processes on both halves of the mandible appeared the same or were asymmetrical. Before the study began, a data collection form was designed, and observations were added to it manually to ensure that all data followed the same format. Next, the data was placed into Microsoft Excel to organize it briefly, then analyzed by using SPSS version 20.0 (IBM Corp., Armonk, NY). The results are mostly shown as percentages and frequencies for each morphological category. Additionally, Chi-square analysis was used to check for any relationship between the side on which a trait is located and its morphology. A p-value of less than 0.05 showed that there was a significant effect.

## Results:

The present work focused on analyzing 80 adult dry human mandibles to see how the shape of the coronoid process varies. The inductions were undertaken on both sides in each jaw, so all 160 coronoid processes were reviewed. The types of morphologies were divided into triangular, rounded and hook-shaped groups. The triangular type was observed most frequently among the 160 coronoid processes studied, occurring in 76 cases (47.5%), followed by the rounded type in 56 cases (35%) and the hook-shaped version in 28 cases (17.5%). A breakdown of the distribution of each morphological type is offered in Table 1 (see PDF). In terms of laterality, bilateral symmetry in coronoid process shape was found in 66 mandibles (82.5%), while asymmetry was seen in 14 mandibles (17.5%). The distribution of symmetrical and asymmetrical mandibles is summarized in Table 2 (see PDF). Further statistical analysis using the Chi-square test did not reveal any significant association between the side (right or left) and the type of coronoid process (p > 0.05), indicating that laterality did not influence morphological variation. These findings highlight the predominance of the triangular type and the general trend of bilateral symmetry among the study sample (Tables 1 and 2 - see PDF).

## Discussion:

The coronoid process plays a crucial role in the biomechanics of mastication by serving as an attachment point for the temporalis muscle. This anatomical feature is not only vital for normal jaw function but also holds significant relevance in the fields of maxillofacial surgery, forensic science, and anthropology [1, 3]. The present study aimed to analyze the morphological variations of the coronoid process in adult human mandibles. The results indicated three predominant morphological types: triangular, rounded, and hook-shaped, with the triangular form being the most common at 47.5%, followed by the rounded and hook-shaped types. These findings are consistent with patterns observed in previous literature [4, 5]. The relatively high occurrence of the rounded type (35%) may suggest a correlation between mandibular morphology and functional factors such as masticatory load and dietary habits [8, 9]. Hook-shaped coronoid processes were less frequently observed (17.5%) and did not appear to significantly affect mandibular function [10]. Symmetry of the coronoid process was also a notable finding, with bilateral symmetry present in 82.5% of samples. This supports the idea of consistent morphological development, potentially influenced by genetic and developmental factors [11, 12-13]. Multiple factors can influence the shape of the coronoid process, including the mechanical action of the temporalis muscle, dietary habits, age-related changes and ethnic variation [14, 15-16]. Generally, greater muscular activity is associated with more pronounced triangular projections, while a less prominent or rounded shape may indicate reduced muscle use or advanced age [17]. Clinically, understanding the morphology of the coronoid process is essential for surgical procedures such as coronoidectomy, which is performed to address conditions like temporomandibular joint ankylosis [18]. Additionally, the coronoid process serves as a donor site in maxillofacial reconstructive surgeries, particularly for repairing the orbital floor and zygomatic arch, making knowledge of its shape and volume crucial for operative planning [19, 20]. From a forensic standpoint, the morphology of the coronoid process can aid in identification and sex estimation. However, in this study, demographic data such as sex were not collected, which limits the interpretive scope regarding sexual dimorphism. Future investigations involving sexed and age-documented skeletal remains are recommended to enhance the understanding of morphological differences across populations [21].

## Conclusion:

Our data is in agreement with previous studies by showing that triangular types of coronoid processes are most common, while the next most frequent are rounded and hook-shaped forms. There is strong consistency in how the lower jaw develops due to its high rate of bilateral symmetry. Knowledge on these variations is crucial for doctors, surgeons, anthropologists, and forensic investigators.
